# Refinements in the reconstruction of bisphosphonate-related osteonecrosis of the jaw

**DOI:** 10.1016/j.jpra.2022.11.004

**Published:** 2022-12-01

**Authors:** Taku Maeda, Yuhei Yamamoto, Akira Satoh, Toshihiko Hayashi

**Affiliations:** a Department of Plastic and Reconstructive Surgery, Faculty of Medicine and Graduate School of Medicine, Hokkaido University, Kita-15 Nishi-7, Kita-ku, Sapporo City, Hokkaido 060-8638, Japan; b Department of Oral Diagnosis and Medicine, Graduate School of Dental Medicine, Hokkaido University, Kita 13, Nishi 7, Kita-ku, Sapporo City, Hokkaido 060-8586, Japan; c Department of Plastic and Reconstructive Surgery, Graduate School of Medicine, Asahikawa Medical University, 2-1-1-1, Midorigaoka higashi, Asahikawa City, Hokkaido 078-8510, Japan

**Keywords:** Bisphosphonate-related osteonecrosis of the jaw, Plate fixation, Microsurgical reconstruction, Progressive, Maxillary, Mandibular

## Abstract

The recommended treatment strategy for stage 3 bisphosphonate-related osteonecrosis of the jaw (BRONJ) is currently rigid plate fixation without bone reconstruction. However, a recent systematic review indicated the utility of microsurgical reconstruction after resection of BRONJ. Several types of flaps have been described but their applications are controversial. Here we present a detailed reconstruction plan for obtaining better outcomes in patients with maxillary and mandibular BRONJ. Given that progressive maxillary BRONJ is often invasive to the skin, including the eyelid, leading to functional loss such as leakage of discharge and ectropion, several revision surgeries are needed to increase the volume in the defect after the free flap transfer. For progressive mandibular BRONJ, hemi-mandibulectomy to subtotal mandibulectomy with an adequate margin from the necrotic bone is necessary, followed by a well-designed fibular free flap.

## Introduction

The recommended treatment strategy for stage 3 bisphosphonate-related osteonecrosis of the jaw (BRONJ) is currently rigid plate fixation without bone reconstruction.^1^ However, bone reconstruction after resection of a stage 3 defect could have a more favorable outcome with better patient satisfaction. A recent systematic review summarized case reports and series of microsurgical reconstruction after resection of BRONJ using a vascularized fibular free flap.^2^ Cases of reconstruction with an iliac crest free flap were also described. Although the exact indications for each type of free flap have yet to be established, it is widely accepted that vascularized free flaps produce better outcomes than rigid plate fixation without bone reconstruction.

Here we report two cases of BRONJ in which we aimed to achieve a better outcome by using free flaps for reconstruction of the maxilla and mandible.

## Case report 1

**Figure 1:**
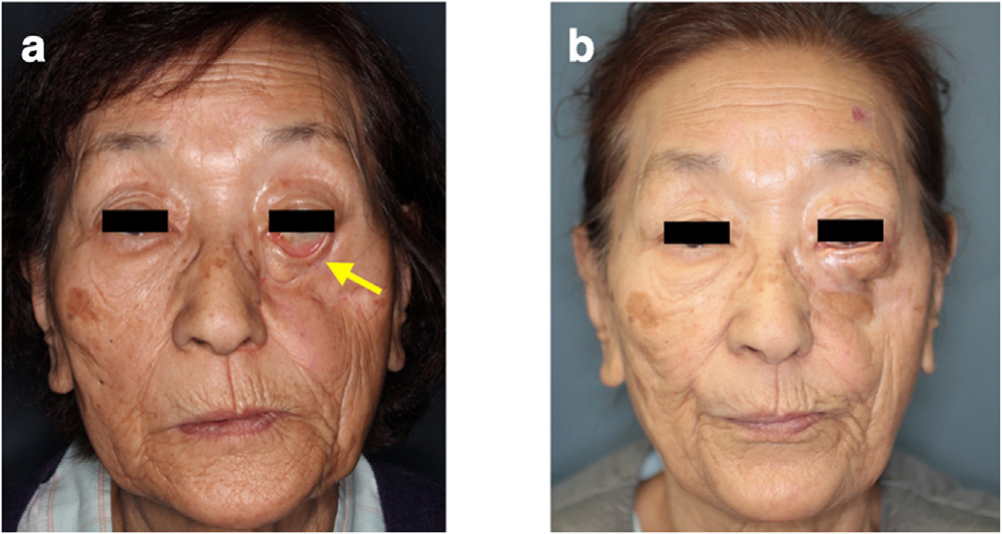
(a) Clinical photographs showing the preoperative appearance of the face. The yellow arrow indicates an ectropion. (b) Clinical photograph obtained 1 year and 2 months after the revision surgery.

A 76-year-old woman with a history of metastatic breast carcinoma was admitted to our department after developing a bone metastasis in a rib following surgical resection of the primary tumor. She was started on intravenous zoledronic acid. Three years and 11 months after initiation of zoledronic acid, she was referred to the department of oral diagnosis and medicine at our hospital for spontaneous tooth prolapse. After confirming that there was no recurrence of breast carcinoma, zoledronic acid was stopped after a total treatment duration of 5 years and 6 months (4 mg every 4 weeks, for a total dose of 264 mg) . However, by this time, necrotic bone had extended to the malar bone, palate, and posterior wall of the maxillary sinus. The necrotic bone in the left maxilla was removed under general anesthesia. Although the BRONJ did not progress further, she had severe lower eyelid ectropion (Figure 1a), an oral fistula, and a depression deformity of the left cheek.

A radial free forearm flap was placed in the left maxilla to close the fistula in the mouth and a non-vascularized rib graft was placed at the ridge of the orbital floor to support the lower eyelid; however, the severe ectropion remained. Revision surgery was performed using a local flap 8 months after the first operation. Although the patient still has a depression in the cheek, the fistula has closed and the ectropion was only slight at 1 year and 2 months after the second operation (Figure 1b).

## Case report 2

**Figure 2:**
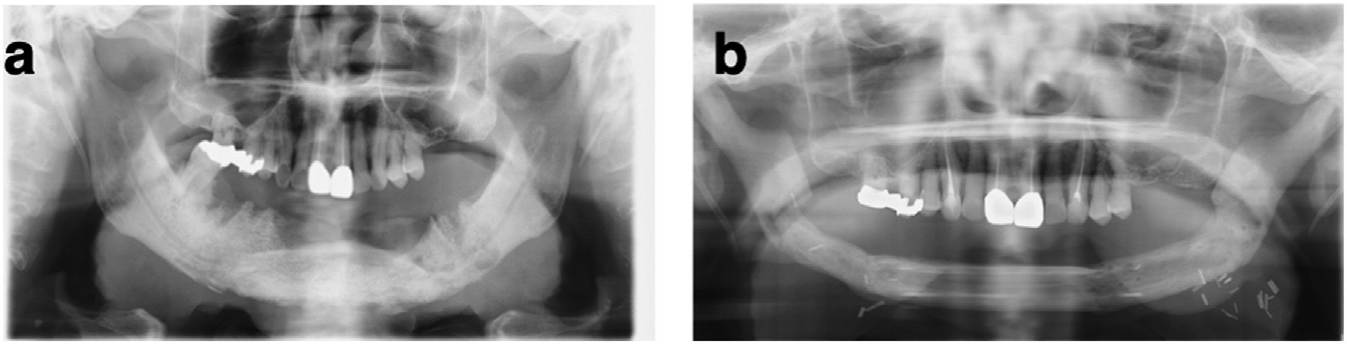
Clinical photographs showing (a) Preoperative radiograph of the face. (b) Postoperative radiograph of the face.

A 73-year-old man who had been diagnosed with prostate cancer 7 years earlier underwent orchiectomy but developed multiple bone metastases, for which he was started on zoledronic acid 4 mg intravenously once a month; 2 years later, after receiving a total dose of 108 mg, he presented to the dental clinic with pain and discharge in the gingiva of a mandibular tooth. Endodontic treatment was commenced but there was no improvement. A year later, the patient consulted the department of oral diagnosis and medicine at our hospital and was diagnosed with stage 2 BRONJ. A small area of exposed bone was noted and zoledronic acid was stopped. However, the area of exposed bone slowly increased and his symptoms worsened. Two years after the initial diagnosis of stage 2 BRONJ, he underwent surgery for exposed mandibular bone on both sides of the mouth and three orocutaneous fistulas of the mandible (BRONJ stage 3; Figure 2a).

Bilateral segmental mandibulectomy was performed and the mandible was reconstructed using a vascularized fibular free flap. The fibular bone graft was reshaped in three pieces to match the shape of the mandible with maintenance of vascularization and fixed with a total of eight plates. The surface of the skin flap was partially removed and used to cover both the internal and external defects. At 2 years and 8 months after surgery, the patient is well with no evidence of recurrence of BRONJ. The shape of the mandible was maintained after radical resection of the mandibular bone. Subsequently, formation of a bone bridge was confirmed on follow-up radiography (Figure 2b).

## Discussion

Patients with stage 3 BRONJ have extensive damage to soft tissue and bone that requires reconstruction using a method that produces the best possible functional and esthetic outcome. The cases reported here have two important clinical lessons. The first point is that because maxillary BRONJ is often invasive to the skin, including the eyelid, and leads to functional loss, including leakage of discharge and ectropion several revision surgeries are necessary after the free flap transfer to increase the volume in the defect. The second point is that hemi-mandibulectomy to subtotal mandibulectomy with an adequate margin from the necrotic bone is necessary for mandibular BRONJ. A carefully designed free fibular flap is very useful for reconstructing a large defect.

There are few reports on patients with refractory BRONJ treated with a microvascular free flap.^3^^,^^4^ Although radial forearm, anterolateral thigh, and iliac crest flaps are typically used, there have been some recent reports demonstrating the success of osteocutaneous fibula flap reconstructions in patients with BRONJ.^3^^,^^5^ These reports have stressed the importance of radical resection followed by free flap reconstruction. but whether or not this treatment strategy produces better outcomes in patient with BRONJ is unclear.

The maxilla appears to be less frequently affected by BRONJ than the mandible.^6^^,^^7^ According to a nationwide survey in Japan, BRONJ was twice as common in the mandible as in the maxilla.^8^ Therefore, recommendations for the treatment of BRONJ in the maxilla are not yet established. Contracture of tissue associated with BRONJ in the maxilla is often associated with ectropion, causing pain, lacrimation, and discomfort. Although soft tissue flaps are essential for maxillary reconstruction,^4^ an additional reconstruction strategy is necessary. In the first case presented here, a radial forearm flap was used to fill the space in the maxilla and oral cavity, and the ectropion was repaired by transfer of cartilage and a local flap for the lower eyelid. Supportive tissue such as cartilage was useful to maintain the height of the lower eyelid rim. Although performing the free flap surgery is the first priority, subsequent revision surgeries should be considered depending on the patient's presenting complaint.

Microvascular replacement of bone is preferred to bridging mandibular plates for reconstruction of defects when segmental mandibulectomy is performed to treat mandibular BRONJ.^9^ Vascularized bone graft reconstruction with a fibular free flap is feasible and has a high success rate in terms of bony union and fistula closure in advanced cases. Detailed preoperative planning is important, and the first step should be resection of the damaged tissue with an adequate margin. The fibular bone needs to be reshaped to match the original shape of the mandible on a case-by-case basis. Adequate resection and a well-designed reshaped fibular free flap are essential.

## Statements & Declarations

### Funding

The authors declare that no funds, grants, or other support were received during the preparation of this manuscript.

## Consent to participate

Informed consent was obtained from all individual participants included in the study.

## Consent to publish

The authors affirm that human research participants provided informed consent for publication of the images in Figure(s) 1a,1b.

## Ethical approval

Not required.

## CRediT authorship contribution statement

**Taku Maeda:** Conceptualization, Data curation, Investigation, Writing – original draft. **Yuhei Yamamoto:** Writing – review & editing. **Akira Satoh:** . **Toshihiko Hayashi:** Supervision, Writing – review & editing.

## Competing Interests

The authors have no relevant financial or non-financial interests to disclose.

## References

[1] Ruggiero S.L., Dodson T.B., Assael L.A., Landesberg R., Marx R.E., Mehrotra B. (2009). American Association of Oral and Maxillofacial Surgeons position paper on bisphosphonate-related osteonecrosis of the jaws–2009 update. J Oral Maxillofac Surg.

[2] Sacco R., Sacco G., Acocella A., Sale S., Sacco N., Baldoni E. (2011). A systematic review of microsurgical reconstruction of the jaws using vascularized fibula flap technique in patients with bisphosphonate-related osteonecrosis. J Appl Oral Sci.

[3] Hanasono M.M., Militsakh O.N., Richmon J.D., Rosenthal E.L., Wax M.K. (2013). Mandibulectomy and free flap reconstruction for bisphosphonate-related osteonecrosis of the jaws. JAMA Otolaryngol Head Neck Surg.

[4] Mucke T., Jung M., Koerdt S., Mitchell D.A., Loeffelbein D., Kesting M.R. (2016). Free flap reconstruction for patients with bisphosphonate related osteonecrosis of the jaws after mandibulectomy. J Craniomaxillofac Surg.

[5] Seth R., Futran N.D., Alam D.S., Knott P.D. (2010). Outcomes of vascularized bone graft reconstruction of the mandible in bisphosphonate-related osteonecrosis of the jaws. Laryngoscope.

[6] Ruggiero S.L., Mehrotra B., Rosenberg T.J., Engroff S.L. (2004). Osteonecrosis of the jaws associated with the use of bisphosphonates: a review of 63 cases. J Oral Maxillofac Surg.

[7] Bamias A., Kastritis E., Bamia C. (2005). Osteonecrosis of the jaw in cancer after treatment with bisphosphonates: incidence and risk factors. J Clin Oncol.

[8] Shibahara T., Morikawa T., Yago K., Kishimoto H., Imai Y., Kurita K. (2018). National survey on bisphosphonate-related osteonecrosis of the jaws in Japan. J Oral Maxillofac Surg.

[9] Head C., Alam D., Sercarz J.A. (2003). Microvascular flap reconstruction of the mandible: a comparison of bone grafts and bridging plates for restoration of mandibular continuity. Otolaryngol Head Neck Surg.

